# Expression and prognostic significance of the PD‐1/PD‐L1 pathway in AIDS‐related non‐Hodgkin lymphoma

**DOI:** 10.1002/cam4.7195

**Published:** 2024-04-13

**Authors:** Han Zhao, Shaohang Cai, Yanhua Xiao, Muye Xia, Hongjie Chen, Zhiman Xie, Xiaoping Tang, Haolan He, Jie Peng, Juanjuan Chen

**Affiliations:** ^1^ Department of Infectious Diseases, Nanfang Hospital Southern Medical University Guangzhou China; ^2^ Infectious Diseases Center, Guangzhou Eighth People's Hospital Guangzhou Medical University Guangzhou China; ^3^ Pathology department, Guangzhou Eighth People's Hospital Guangzhou Medical University Guangzhou China; ^4^ Guangxi AIDS Clinical Treatment Center, the Fourth People's Hospital of Nanning Nanning China

**Keywords:** acquired immunodeficiency syndrome, Epstein–Barr virus, non‐Hodgkin lymphoma, programmed cell death protein 1, regulatory T cells

## Abstract

**Objective:**

Immune tolerance and evasion play a critical role in virus‐driven malignancies. However, the phenotype and clinical significance of programmed cell death 1 (PD‐1) and its ligands, PD‐L1 and PD‐L2, in aggressive acquired immunodeficiency syndrome (AIDS)‐related non‐Hodgkin lymphoma (AR‐NHL) remain poorly understood, particularly in the Epstein–Barr virus (EBV)‐positive subset.

**Methods:**

We used in situ hybridization with EBV‐encoded RNA (EBER) to assess the EBV status. We performed immunohistochemistry and flow cytometry analysis to evaluate components of the PD‐1/PD‐L1/L2 pathway in a multi‐institutional cohort of 58 patients with AR‐NHL and compared EBV‐positive and EBV‐negative cases.

**Results:**

The prevalence of EBV^+^ in AR‐NHL was 56.9% and was associated with a marked increase in the expression of PD‐1/PD‐L1/PD‐L2 in malignant cells. Patients with AR‐NHLs who tested positive for both EBER and PD‐1 exhibited lower survival rates compared to those negative for these markers (47.4% vs. 93.8%, *p* = 0.004). Similarly, patients positive for both EBER and PD‐L1 also demonstrated poorer survival (56.5% vs. 93.8%, *p* = 0.043). Importantly, PD‐1 tissue‐expression demonstrated independent prognostic significance for overall survival in multivariate analysis and was correlated to elevated levels of LDH (*r* = 0.313, *p* = 0.031), increased PD‐1^+^ Tregs (*p* = 0.006), and robust expression of EBER (*r* = 0.541, *p* < 0.001) and PD‐L1 (*r* = 0.354, *p* = 0.014) expression.

**Conclusions:**

These data emphasize the importance of PD‐1‐mediated immune evasion in the complex landscape of immune oncology in AR‐NHL co‐infected with EBV, and contribute to the diagnostic classification and possible definition of immunotherapeutic strategies for this unique subgroup.

## 1 INTRODUCTION

Individuals living with HIV (PLWH) have a higher lifetime risk of developing certain cancers. Kaposi's sarcoma, non‐Hodgkin lymphoma (NHL), and invasive cervical cancer are considered AIDS‐defining cancers.^1^ HIV contributes to carcinogenesis and lymphomagenesis through direct viral activity, immunosuppression, immune dysregulation, chronic antigenic stimulation, and possibly the secretion of viral proteins.^2^ In the combination antiretroviral therapy (cART) era, aggressive acquired immunodeficiency syndrome (AIDS)‐related NHL (AR‐NHL) remains one of the most common malignancies.^2^, ^3^ These patients lack comprehensive characterization, exhibit limited responses to standard chemotherapeutic approaches, and are associated with Epstein–Barr virus (EBV) co‐infections.^4^, ^5^ Despite the tremendous advances in cancers and HIV care in recent decades, patients with HIV‐associated cancers continue to experience poor outcomes in contrast to the non‐immunosuppressed population, highlighting distinct oncogenic mechanisms and immune dysregulation.^6^ However, the prevalence and pathogenesis of EBV^+^ AR‐NHL remain unclear in China and optimal management strategies are unknown.

In general, EBV infections trigger robust cell immunity and activate intracellular signaling, which contribute to immune tolerance and evasion of virus‐associated lymphoma cells.^7^ Most EBV‐driven lymphomas have high levels of programmed cell death protein 1 (PD‐1)/programmed death‐ligand 1 (PD‐L1) expression, typically due to latent membrane protein‐1‐mediated activation of the CD274 JAK/STAT‐dependent promoter or AP‐1‐associated enhancer activity.^8^ PD‐1/PD‐L1 overexpression serves as an indicator of a poor prognosis in aggressive lymphomas.^5^ Several clinicopathological features, including chromosome 9p amplification/translocation, EBV infection, and an activated B‐cell‐like phenotype, have been associated with PD‐1/PD‐L1 expression in lymphomas.^9^, ^10^, ^11^ In fact, immune checkpoint inhibitors have revolutionized cancer treatment not only for Hodgkin lymphoma but also for various solid tumors and other hematologic malignancies in clinical practice.^12^, ^13^ In addition, the amount of target protein in tumor tissue and/or in the microenvironment is a factor contributing to their therapeutic effectiveness.^8^

However, most of the available data has been evaluated in HIV‐uninfected patients and might not be representative of HIV populations. Studies evaluating PD‐1/PD‐L1 expression and its clinical significance in AR‐NHL are not available for the Chinese population.

In this multicenter real‐world study, our objective was to explore EBV status and PD‐1/PD‐L1/PD‐L2 expression in a cohort of patients with AR‐NHL, with the objective of identifying histopathological characteristics and defining prognostic implications. These findings offer a basis for considering immunotherapy as a potential treatment approach.

## 2 MATERIALS AND METHODS

### 2.1 Patients and clinical data

Between January 2011 and December 2021, we enrolled 58 of 237 patients with de novo AR‐NHL from Nanfang Hospital, Guangzhou Eighth People's Hospital, and the Fourth People's Hospital of Nanning (Figure S1). This research received approval from the Ethics Committees of Nanfang Hospital (NFEC‐2021‐178), Guangzhou Eighth People's Hospital (202210222), and the Fourth People's Hospital of Nanning ([2019]39). All participants provided written informed consent in accordance with the World Medical Association Declaration of Helsinki. These patients had available formalin‐fixed paraffin‐embedded (FFPE) and peripheral blood samples. NHL diagnosis was confirmed in accordance with the 2008 or 2016 World Health Organization classification.^14^, ^15^ Clinical information was obtained from electronic and/or paper‐based medical records. The optimal cutoff value for the CD4/CD8 ratio was determined to be 0.41 through ROC analysis, as described in our previous study.^16^ Overall survival (OS) was defined as the time from the initial diagnosis of AR‐NHL to patient death or the last follow‐up.

### 2.2 Immunohistochemistry and in situ hybridization studies

We constructed tissue microarrays (TMA) (Method S1) and used EBV‐encoded RNA (EBER) to assess EBV status in 58 cases. Chromogenic in situ hybridization (ISH) for EBER was detected with a fluorescein‐conjugated EBER RISH kit (ISH‐7001, Zhongshan Goldenbridge Biotechnology Co., China) following the manufacturer's protocol. A Burkitt lymphoma with known positivity for EBV was used as a positive control and hybridization without a probe was performed as a negative control (Figure S2A). Immunohistochemistry (IHC) was performed following established protocols (Method S1),^17^ covering CD20, PAX5, CD3, CD4, CD5, CD8, CD10, CD21, CD22, CD19, CD30, CD38, CD56, CD138, CD79a, ALK, BCL6, BCL2, MUM1, and MYC. Various analyses were performed or reviewed using FFPE tissue sections, utilizing an automated immunostainer (Dako Omnis and Roche Benchmark XT). Heat‐induced antigen retrieval was used for all tissue sections.

The expression of PD‐1/PD‐L1/L2 on lymphoma cells was evaluated in 48 patients using IHC on available TMA (Method S1). This evaluation used antibodies targeting PD‐1 (Abcam), PD‐L1 (22C3, Dako), or PD‐L2 (Abcam), in addition to the B cell‐specific marker PAX5 (Abcam) (Figure S2B). Positive staining for membranous PD‐L1 and PD‐L2 was defined when the immunoreactivity score (IRS) exceeded 5% of tumor cells (TCs) or tumor‐infiltrating lymphocytes (TILs). Additionally, a positive score was assigned to PD‐1 staining when observed in more than 5% of TIL cells (Method S1).^18^, ^19^, ^20^ All PD‐1/PD‐L1/L2 IHC results were independently reviewed and scored by two hematopathologists, ensuring a blinded evaluation to pathological types.

### 2.3 Flow cytometry

Multiparameter flow cytometry (FCM) data from peripheral blood mononuclear cells (PBMC) of 26 available cases were acquired using a Cytek Aurora flow cytometer (Cytek Biosciences, USA). Staining was carried out according to standard protocols (Method S2) to determine the expression of CD3, CD4, CD8, CD134, PD‐1, CD25, CD127, and CD45RA. Data analysis was performed on compensated data using FlowJo V10.8.1 software (TreeStar, Oregon, USA). All experiments were conducted in a biosafety laboratory. For prognosis analysis, the median% expression of PD‐1 was used as the threshold.

### 2.4 Statistical analysis

Statistical analyses and graphical rendering were performed with SPSS software (version 21.0; Chicago, IL, USA) and GraphPad Prime 9, respectively. Quantitative variables were described as median (interquartile range, IQR). The chi‐squared test was used for descriptive statistical analyses of categorical data. Bivariate correlations among variables were conducted using Pearson's correlation test. Linear correlation analysis and the Mann–Whitney *U*‐test were used to examine the relationship between PD‐1 TISSUE expression and other factors. Survival curves were generated using the Kaplan–Meier method and curve comparisons were performed using the log‐rank test. Univariate Cox regression was used to calculate hazard ratios, multivariate Cox regression was applied to variables with *p* values up to 0.05, and the proportionality assumption for the Cox analysis has been confirmed to be met. All *p* values were two‐sided and *p* < 0.05 was considered statistically significant.

## 3 RESULTS

### 3.1 Clinical characteristics and clinicopathological features

**FIGURE 1:**
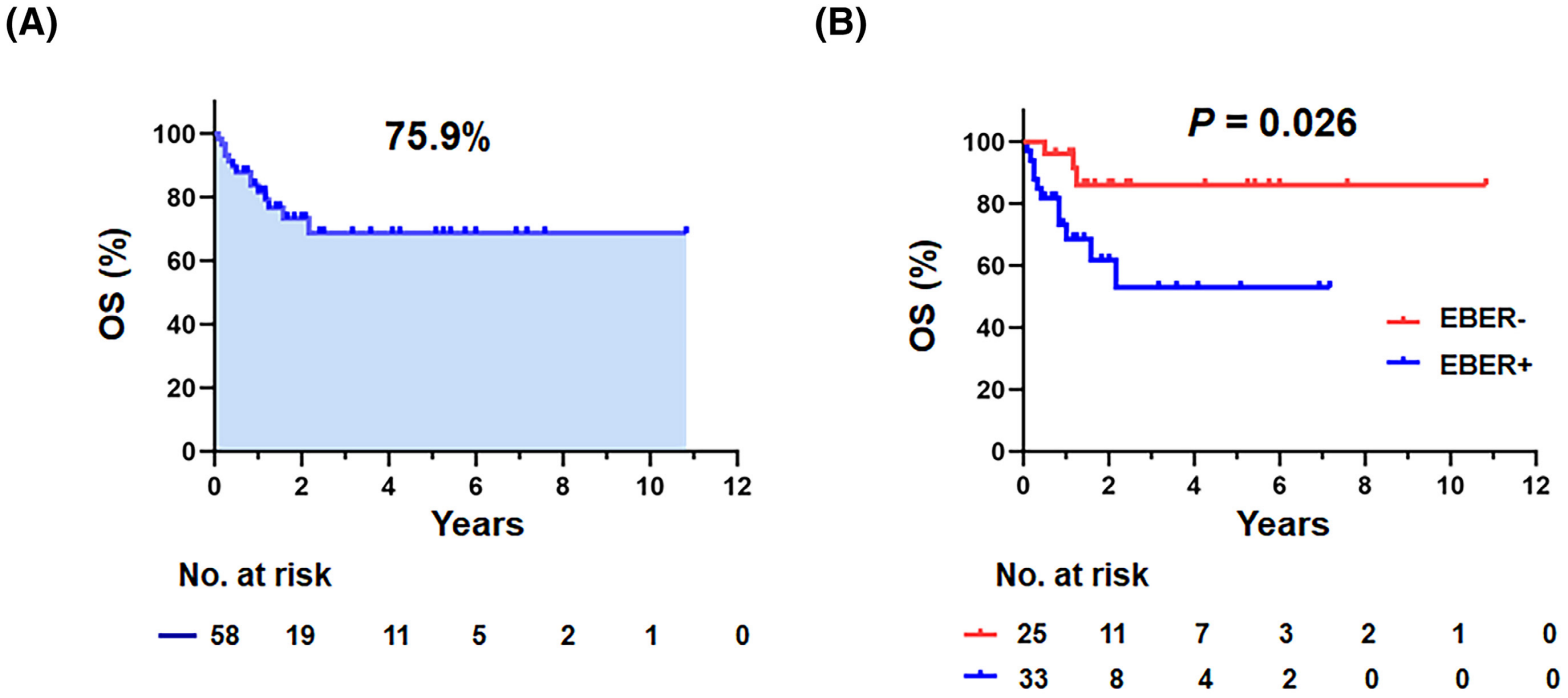
Survival analysis. (A) Overall survival (OS) of AIDS‐related non‐Hodgkin lymphomas (AR‐NHL) in the entire cohort. (B) Kaplan–Meier survival analysis stratified by EBV‐encoded RNA (EBER) status.

Fifty‐eight newly diagnosed patients with AR‐NHL were included in this analysis. Baseline clinical characteristics are summarized in Table 1, and diagnoses included diffuse large B‐cell lymphoma (DLBCL, 60.4%), Burkitt lymphoma (BL, 27.6%), plasmablastic lymphoma (PBL, 3.4%), follicular lymphoma (1.7%), high‐grade B‐cell lymphoma (1.7%), and T‐cell lymphoma (5.2%). The patients had a median age of 47.5 years (IQR 38.5–57.3), and 82.8% were men. At baseline, 19.0% were aged >60 years old, 65.5% had elevated LDH levels, 46.6% had ECOG PS >1, 60.3% were in Stage III/IV, and 67.2% had documented extra‐nodal >1 involvement. Most of the patients (56.9%) had elevated β2‐microglobulin levels, while 32.8% exhibited B symptoms and 32.8% bulky masses (>7.5 cm). OS at a median follow‐up of 15 months (range 1–130) was 75.9% (Figure 1A), with 79.3% of patients receiving chemotherapy. One patient presented with CNS disease at the time of diagnosis, and this patient refused chemotherapy. Twenty‐five (43.1%) patients received CNS prophylaxis with intrathecal methotrexate, cytarabine, and dexamethasone. Among the 58 patients, 12 (20.7%) had complete response (CR) and 20 had partial response (PR) status (34.5%) (Table S1).

**TABLE 1:** Clinical and pathological characteristics of AIDS‐related non‐Hodgkin lymphoma (*n* = 58).

Variable	Total (*N* = 58)	EBER^−^ (*n* = 25)	EBER^+^ (*n* = 33)	*p*
Male sex, *n* (%)	48 (82.8)	22 (45.8)	26 (54.2)	0.490
Age (median, IQR)	47.5 (38.5–57.3)			0.098
≤40, *n* (%)	17 (29.3)	11 (64.7)	6 (35.3)	
41–60, *n* (%)	30 (51.7)	12 (40.0)	18 (60.0)	
61–75, *n* (%)	10 (17.2)	2 (20.0)	8 (80.0)	
≥75, *n* (%)	1 (1.7)	0 (0)	1 (100.0)	
AIDS before lymphoma diagnosis, *n* (%)	15 (25.9)	5 (33.3)	10 (66.7)	0.546
Lymphoma subtype, *n* (%)				0.629
B‐cell lymphoma	55 (94.8)	24 (43.6)	31 (56.4)	
DLBCL	35 (60.4)	16 (45.7)	19 (54.3)	
GCB type	21 (60.0)	8 (38.1)	13 (61.9)	0.317
Non‐GCB type	14 (40.0)	8 (57.1)	6 (42.9)	
BL	16 (27.6)	6 (37.5)	10 (62.5)	
PBL	2 (3.4)	1 (50.0)	1 (50.0)	
FL	1 (1.7)	0 (0)	1 (100.0)	
HGBL	1 (1.7)	1 (100.0)	0 (0)	
T‐cell lymphoma	3 (5.2)	1 (33.3)	2 (66.7)	
ALK‐negative ALCL	1 (1.7)	1 (100.0)	0 (0)	
NKTL	1 (1.7)	0 (0)	1 (100.0)	
PTCL	1 (1.7)	0 (0)	1 (100.0)	
ECOG PS >1, *n* (%)	27 (46.6)	12 (44.4)	15 (55.6)	1.000
Elevated LDH, *n* (%)	38 (65.5)	14 (36.8)	24 (63.2)	0.265
Ann Arbor stage III/IV, *n* (%)	35 (60.3)	16 (45.7%)	19 (54.3%)	0.787
Extra‐nodal sites >1, *n* (%)	39 (67.2)	13 (33.3)	26 (66.7)	**0.048**
B symptoms present, *n* (%)	19 (32.8)	6 (31.6)	13 (68.4)	0.266
Bulky tumor ≥ 7.5 cm, *n* (%)	19 (32.8)	4 (21.1)	15 (78.9)	**0.024**
β2‐MG, *n* (%)				1.000
Elevated	33 (56.9)	9 (27.3)	24 (72.7)	
Unknown	22 (37.9)			
HIV‐1 RNA (copies/mL), *n* (%)				0.103
Below the limit of quantification	23 (39.7)	12 (52.2)	11 (47.8)	
<1 × 10^5^	17 (29.3)	4 (23.5)	13 (76.5)	
≥1 × 10^5^	10 (17.2)	6 (60.0)	4 (40.0)	
Unknown	8 (13.8)			
CD4^+^ T‐cell count (cells/μL), (median, IQR)	182 (92–304)			0.597
<200, *n* (%)	33 (56.9)	13 (39.4)	20 (60.6)	
≥200, *n* (%)	25 (43.1)	12 (48.0)	13 (52.0)	
CD4/CD8 ratio (median, IQR)	0.29 (0.14–0.44)			1.000
<0.41	40 (69.0)	17 (42.5)	23 (57.5)	
≥0.41	18 (31.0)	8 (44.4)	10 (55.6)	
cART (yes), *n* (%)	55 (94.8)	23 (41.8)	32 (58.2)	0.572
Chemotherapy (yes), *n* (%)	46 (79.3)	21 (45.7)	25 (54.3)	0.526
Ki‐67, *n* (%)				**0.020**
30%–80%	16 (27.6)	11 (68.8)	5 (31.2)	
>80%	42 (72.4)	14 (33.3)	28 (66.7)	
CD20, *n* (%)				0.632
Positive	51 (87.9)	22 (43.1)	29 (56.9)	
Negative	4 (91.4)	1 (25.0)	3 (75.0)	
Unknown	3 (3.4)			

Bold value indicates *p* < 0.05. Abbreviations: AIDS, acquired immunodeficiency syndrome; ALCL, anaplastic large cell lymphoma; ALK, anaplastic lymphoma kinase; BL, Burkitt lymphoma; cART, combined antiviral therapy.; DLBCL, diffuse large B‐cell lymphoma; EBER, EBV‐encoded RNA; ECOG PS, Eastern Cooperative Oncology Group performance status; FL, follicular lymphoma; GCB, germinal‐center B‐cell‐like; HGBL, high‐grade B‐cell lymphoma; HIV, human immunodeficiency virus; IQR, interquartile range; LDH, lactate dehydrogenase; NKTL, nature kill T cell lymphoma; PBL, plasmablastic lymphoma; PTCL, peripheral T‐cell lymphoma; β2‐MG, β2‐microglobulin.

At the time of the initial lymphoma diagnosis, the median CD4^+^ T count was 182 cells/μL (IQR 92–304). Fifty‐five (94.8%) patients received combined antitumor therapy and cART (Table 1; Table S1). A plasma HIV‐1 viral load >100,000 copies/mL was present in 10 of 50 (20.0%) patients. At the time of lymphoma diagnosis, 15 of 58 (25.9%) patients had a history of HIV and had been on cART for more than 3 months. Among these 15 patients, 11 (73.3%) had a CD4^+^ T‐cell count >200 cells/μL, and 12 (80.0%) had an HIV‐1 viral load below the quantification limit.

In AIDS‐related B‐cell lymphoma patients, a high IPI score (3–5) was observed in 27 (49.1%) patients. Additionally, double‐expressor lymphoma (DEL), double‐hit lymphoma (DHL), triple‐expressor lymphoma (TEL), and triple‐hit lymphoma (THL) were found in 50%, 16.6%, 38.9%, and 5.6% of cases, respectively (Table S2).

### 3.2 Prevalence and prognosis of EBER status in AR‐NHL

EBER status was positive in 33 patients with AR‐NHL (56.9%, Table 1), which was substantial, higher than that of ordinary NHL (5–15%).^5^ Patients with EBER^+^ AR‐NHL, compared to patients with EBER^−^ AR‐NHL, exhibited higher frequencies of bulky tumors (78.9% vs. 21.1%, *p* = 0.024) and a greater Ki‐67 proliferation index (66.7% vs. 33.3%, *p* = 0.020), in accordance with previous research.^5^ Furthermore, patients with EBER^+^ status had a higher incidence of extra‐nodal involvement (66.7% vs. 33.3%, *p* = 0.048), primarily affecting the gastrointestinal tract, skin, and bone marrow (Table 1). Of note, EBER^+^ AR‐NHL demonstrated inferior survival compared to patients with EBER^−^ AR‐NHL, with a hazard ratio for death of 3.872 (95% CI: 1.073–13.974, *p* = 0.026, Figure 1B).

### 3.3 Increased expression of PD‐1/PD‐L1/PD‐L2 in EBER ^+^ AR‐NHLs

**FIGURE 2:**
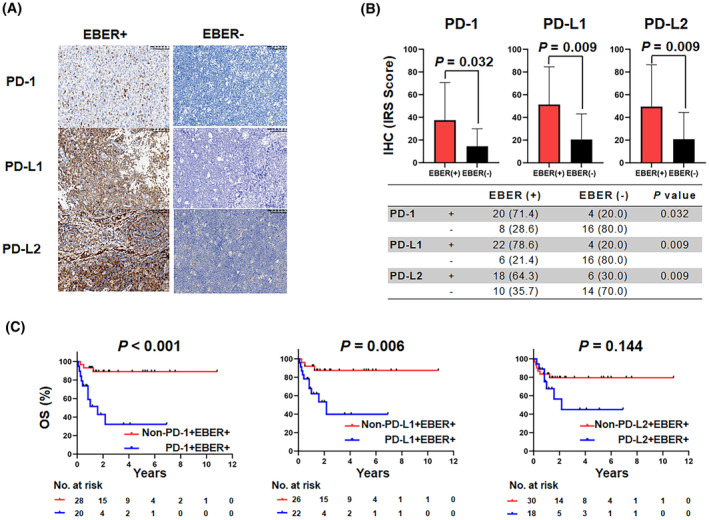
AR‐NHLs infected with EBV harbored high PD‐1/PD‐L1/PD‐L2 expression, associated with poorer outcomes. (A) Representative IHC images of PD‐1, PD‐L1, and PD‐L2 in the EBER^+^ and EBER^−^ AR‐NHL subgroups (200×). (B) Different immunoreactivity score (IRS) for PD‐1, PD‐L1, and PD‐L2 between EBER^+^ and EBER^−^ AR‐NHL tissues. (C) Overall survival of patients with AR‐NHL stratified by EBER, PD‐1, PD‐L1, and PD‐L2, determined by Kaplan–Meier survival analyses.

We further investigated the clinical importance of the programmed cell death axis in the context of AR‐NHL to uncover the correlation between immune evasion and viral etiology, such as EBV.^12^ PD‐L1 showed positive expression in 26 of 48 AR‐NHL patients (54.2%) with available tissue specimens, while PD‐1 and PD‐L2 both showed positive expression rates of 50% (Figure 2A). In particular, robust PD‐L1 membrane staining was observed in most patients with EBER^+^ AR‐NHL (22/28 [78.6%], *p* = 0.009, Figure 2B). Similarly, EBER^+^ AR‐NHL showed strong staining for PD‐1 (20/28 [71.4%], *p* = 0.032) and PD‐L2 (18/28 [64.3%], *p* = 0.009) in malignant cells (Figure 2B). Impressively, patients with PD‐1^+^ EBER^+^ (*p* < 0.001) and PD‐L1^+^ EBER^+^ (*p* = 0.006) had a significantly worse OS compared to those without PD‐1^+^/PD‐L1^+^ EBER^+^ expression (Figure 2C).

### 3.4 Histological expression of PD‐1 independently predicted survival in patients with AR‐NHLs

**FIGURE 3:**
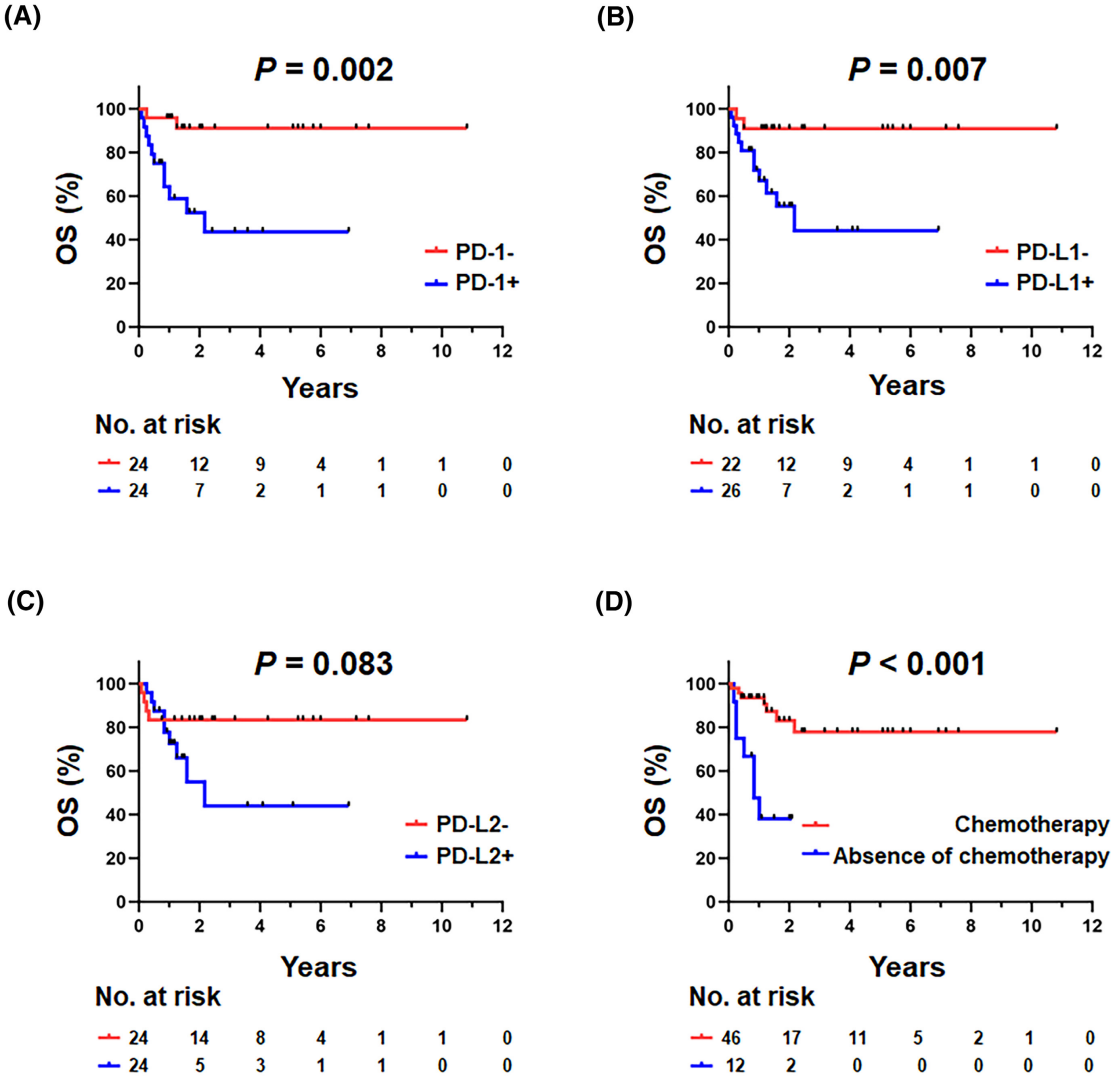
Prognostic significance based on independent predictors and the PD‐1/PD‐L1 pathway in patients with AR‐NHL. Kaplan–Meier curves for OS of (A) PD‐1, (B) PD‐L1, (C) PD‐L2, and (D) chemotherapy status.

In the univariate Cox regression (Table 2), positive expression of PD‐1 (HR = 7.561, *p* = 0.002), and PD‐L1 (HR = 6.189, *p* = 0.007), EBER^+^ (HR = 3.872, *p* = 0.026), IPI score >3 (HR = 3.829, *p* = 0.018), elevated LDH (HR = 10.356, *p* = 0.025), extra‐nodal sites >1 (HR = 4.096, *p* = 0.048), HIV‐1 RNA > 1 × 10^5^ copies/mL (HR = 2.037, *p* = 0.046), B symptoms (HR = 4.901, *p* = 0.002), absence of cART (HR = 4.049, *p* = 0.049), and absence of chemotherapy (HR = 6.767, *p* < 0.001) were individual prognostic factors for the outcome in AR‐NHL patients. Similarly, patients with AR‐NHL with positive expression of PD‐1 (*p* = 0.002) and PD‐L1 (*p* = 0.007), as well as those without chemotherapy (*p* < 0.001), exhibited poorer clinical outcomes in terms of Kaplan–Meier analysis (Figure 3A,B,D). Given the limited size of our cohort, the inclusion of IPI score, PD‐1, PD‐L1, and EBER in the multivariate analysis aimed to ensure more robust results. The histological expression of PD‐1 (HR = 17.656, *p* = 0.028) and an IPI score >3 (HR = 17.449, *p* = 0.016) maintained statistical significance as independent prognostic factors for inferior OS (Table 2).

**TABLE 2:** The prognostic factors associated with overall survival in AIDS‐related non‐Hodgkin lymphoma patients (*n* = 58).

Variables	Univariate analysis		Multivariate analysis	
	HR (95% CI)	*p* Value	HR (95% CI)	*p* Value
PD‐1^+^	7.561 (1.665–34.339)	**0.002**	6.385 (1.200–33.968)	**0.030**
PD‐L1^+^	6.189 (1.361–28.146)	**0.007**	2.776 (0.438–17.579)	0.278
PD‐L2^+^	2.759 (0.835–9.120)	0.083		
EBER^+^	3.872 (1.073–13.974)	**0.026**	1.817 (0.266–12.400)	0.542
IPI score >3	3.829 (1.260–11.634)	**0.018**	6.966 (1.891–25.661)	**0.004**
Male	1.258 (0.281–5.634)	0.763		
Age > 60	2.201 (0.688–7.043)	0.171		
Prior history of HIV	0.761 (0.212–2.737)	0.675		
Lymphoma subtype (DLBCL)	1.295 (0.962–1.743)	0.076		
ECOG PS >1	2.891 (0.953–8.770)	0.050		
Ann Arbor stage III/IV	3.259 (0.901–11.785)	0.056		
Elevated LDH, *n* (%)	10.356 (1.332–80.489)	**0.025**		
Extra‐nodal sites >1	4.096 (0.907–18.488)	**0.048**		
B symptoms (present)	4.901 (1.622–14.812)	**0.002**		
Bulky tumor ≥ 7.5 cm	1.808 (1.625–5.229)	0.268		
Elevated β2‐MG	23.901 (0.001–5.374E5)	0.337		
HIV‐1 RNA ≥1 × 10^5^ copies/mL	2.037 (0.982–4.226)	**0.046**		
CD4^+^ T‐cell count <200 cells/μL	1.280 (0.448–3.656)	0.644		
CD4/CD8 ratio <0.41	1.222 (0.2382–3.911)	0.735		
Absence of cART	4.049 (0.895–18.325)	**0.049**		
Absence of chemotherapy	6.767 (2.237–20.471)	**<0.001**		

Bold value indicates *p* < 0.05. Abbreviations: AIDS, acquired immunodeficiency syndrome; cART, combined antiviral therapy; DLBCL, diffuse large B‐cell lymphoma; EBER, Epstein–Barr virus‐encoded RNA; ECOG PS, Eastern Cooperative Oncology Group performance status; HIV, human immunodeficiency virus; IPI, International Prognostic Index; LDH, lactate dehydrogenase; PD‐1, programmed cell death 1; PD‐L1, programmed cell death ligand 1; PD‐L2, programmed cell death ligand 2; β2‐MG, β2‐microglobulin.

### 3.5 Association of histological PD‐1 with clinical variables in AR‐NHLs

**FIGURE 4:**
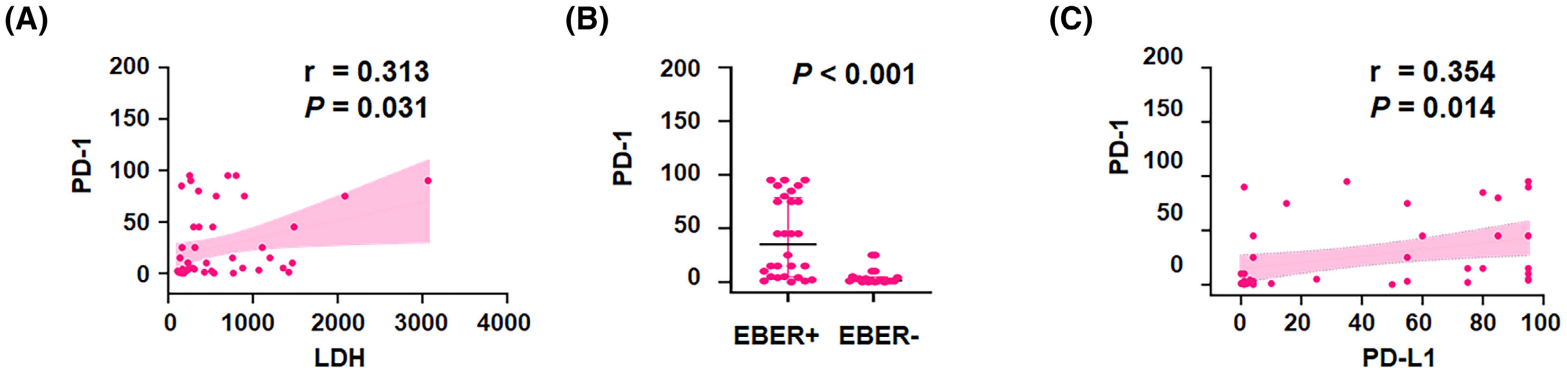
Correlation between PD‐1 and clinical variables in AR‐NHLs. Expression of PD‐1 expression in relation to (A) LDH, (B) EBER, and (C) PD‐L1 levels.

The chi‐squared test (Table 3) identified significant correlations between the histological expression of PD‐1 and elevated LDH levels (*p* = 0.015), high plasma HIV‐1 viral load (*p* = 0.049), EBER status (*p* < 0.001), and PD‐L1 status (*p* = 0.008). Similarly, the IRS for PD‐1 was positively correlated with LDH levels (*r* = 0.313, *p* = 0.031), EBER status (*p* < 0.001), and the IRS for PD‐L1 (*r* = 0.354, *p* = 0.014) in the linear correlation analysis and comparative statistics, as shown in Figure 4A–C, respectively.

**TABLE 3:** Clinical and clinicopathological variables stratified by histological PD‐1 of AIDS‐related non‐Hodgkin lymphoma patients (*n* = 48).

Variables	PD‐1		
	Negative (*n* = 24, %)	Positive (*n* = 24, %)	*p* Value
Male	19 (79.2)	20 (83.3)	1.000
Age > 60 years	2 (8.3)	8 (33.3)	0.072
Prior history of HIV	8 (33.3)	6 (25.0)	0.752
Lymphoma subtype (DLBCL)	16 (66.7)	14 (58.3)	0.909
ECOG PS >1	8 (33.3)	13 (54.2)	0.244
Elevated LDH	11 (45.8)	20 (83.3)	**0.015**
Extra‐nodal sites >1	12 (50.0)	19 (79.2)	0.069
B symptoms (present)	7 (29.2)	10 (41.7)	0.547
Bulky tumor ≥7.5 cm	7 (29.2)	8 (33.3)	1.000
Elevated β2‐MG	11 (100)	16 (88.9)	0.512
HIV‐1 RNA ≥1 × 10^5^ copies/mL	5 (20.8)	3 (12.5)	**0.049**
CD4^+^ T‐cell count <200 cells/μL	12 (50.0)	15 (62.5)	0.561
CD4/CD8 ratio <0.41	15 (62.5)	17 (70.8)	0.760
Absence of cART	1 (4.2)	1 (4.2)	1.000
Absence of chemotherapy	5 (20.8)	7 (29.2)	0.740
EBER^+^	8 (33.3)	20 (83.3)	**<0.001**
PD‐L1^+^	8 (33.3)	18 (75.0)	**0.008**
PD‐L2^+^	9 (37.5)	15 (62.5)	0.148

Bold value indicates *p* < 0.05. Abbreviations: AIDS, acquired immunodeficiency syndrome; cART, combined antiviral therapy; DLBCL, diffuse large B‐cell lymphoma; EBER, Epstein–Barr virus‐encoded RNA; ECOG PS, Eastern Cooperative Oncology Group performance status; HIV, human immunodeficiency virus; LDH, lactate dehydrogenase; PD‐1, programmed cell death 1; PD‐L1, programmed cell death ligand 1; PD‐L2, programmed cell death ligand 2; β2‐MG, β2‐microglobulin.

### 3.6 PD‐1^+^ Tregs were more frequent in PD‐1 IHC‐positive patients and predicted inferior survival

**FIGURE 5:**
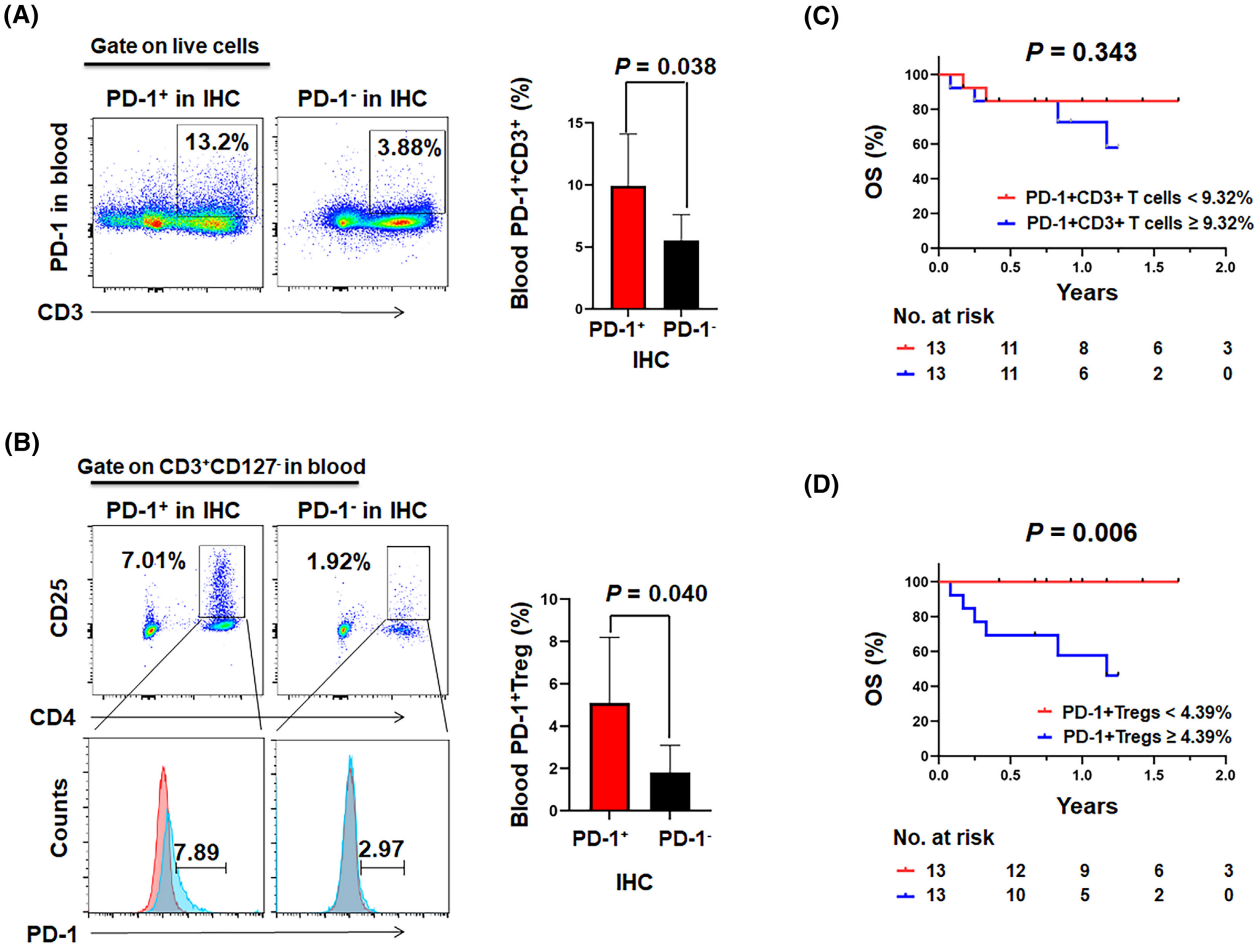
PD‐1^+^ Tregs were more common in PD‐1 IHC‐positive AR‐NHLs patients and correlated with poorer survival. Representative flow cytometry staining (left) and summary (right) of PD‐1 expression in (A) CD3^+^ T cells and (B) Tregs (CD3^+^CD4^+^CD25^+^CD127^−^) in peripheral blood. Kaplan–Meier survival analyses stratified by (C) PD‐1^+^CD3^+^ T cells and (D) PD‐1^+^ Treg.

As histological PD‐1 is an independent prognostic factor in AR‐NHL, our objective was to investigate whether the expression of PD‐1 in histological samples was related to its expression in specific subsets of T cells in the blood. To address this, we analyzed the distribution of PD‐1 in various subsets of T cells using PBMC samples from 26 patients with AR‐NHL, 16 of whom had sufficient tissue for IHC analysis. The FCM analysis revealed a higher percentage of PD‐1^+^ CD3^+^ T cells in PD‐1 IHC‐positive patients (9.94 ± 1.26%) compared to PD‐1 IHC‐negative patients (5.51 ± 0.94%, *p* = 0.028, Figure 5A; Table S3). Furthermore, PD‐1^+^ expressing Tregs (PD‐1^+^ CD3^+^ CD4^+^ CD25^+^ CD127^−^) were notably more abundant in PD‐1 IHC‐positive patients than PD‐1 IHC‐negative patients (5.09 ± 0.93% vs. 1.81 ± 0.57%, *p* = 0.041, Figure 5B; Table S3), but there were no statistically significant differences observed in PD‐1^+^ CD3^+^ CD4^+^ T cells, PD‐1^+^ CD3^+^ CD8^+^ T cells, PD‐1^+^ inhibited T cells, PD‐1^+^ CD3^+^ CD8^+^ inhibited T cells, PD‐1^+^ resting Tregs, and PD‐1^+^ activated Tregs between two groups (Table S3). In Figure 5C, a Kaplan–Meier plot is displayed, there was no significant difference in survival according to PD‐1^+^ CD3^+^ T cells numbers. Furthermore, higher levels of PD‐1^+^ Tregs in peripheral blood were associated with worse OS (*p* = 0.006, Figure 5D).

## 4 DISCUSSION

In our study, a high prevalence of EBV^+^ was identified in patients with AR‐NHL, who displayed aggressive clinical parameters and clinicopathological characteristics, resulting in an inferior prognosis compared to patients with EBV^−^ status. Importantly, PD‐1/PD‐L1 staining of lymphoma cells was strongly correlated with EBV^+^, may account for a worse outcome. It should be noted that PD‐1 served as an independent pathological and molecular prognostic biomarker associated with adverse OS in patients with AR‐NHL, and was associated with PD‐1^+^ Tregs numbers in the peripheral blood. These findings indicated that the immune checkpoint signaling axis contributed to immune escape in EBV^+^ AR‐NHL, highlighting the potential therapeutic value of targeting the PD‐1/PD‐L1 pathway in this rare subset.

EBV^+^ is frequently observed in various AR‐NHL subsets, including DLBCL, PBL, PEL, NK/T cell lymphoma, and angioimmunoblastic T‐cell lymphoma (AITL).^21^ In our study, the prevalence of AR‐DLBCL cells co‐infected with EBV was 54.3%, which is significantly comparable to the rate in the USA (48%)^4^ but higher than in South Africa (16%).^22^ Previous studies in HIV‐infected populations have suggested that approximately 37% of malignancies,^23^, ^24^ 16.0%–48% of DLBCL,^4^, ^22^ 92.0% of PCNSL,^25^ 73.7% of PEL,^26^ and 74%–77.8% of cases of PBL^27^, ^28^ are associated with EBV infection. This prevalence is significantly higher than those observed in HIV‐seronegative individuals (5%–15%).^5^ Furthermore, our data highlighted the distinctive characteristics of EBV^+^ AR‐NHL compared with EBV^−^ cases, including a worse prognosis, a higher frequency of extranodal involvement, a larger tumor, and increased aggressiveness. These findings are consistent with previous studies.^4^, ^25^, ^29^ Advancements in understanding the pathogenesis of NHLs associated with oncogenic viruses could provide valuable treatment targets.

Immune tolerance and evasion are crucial in virus‐driven malignancies.^30^ However, the mechanisms underlying immune escape in EBV^+^ AR‐NHL remain poorly defined. In this study, a significant proportion of EBV^+^ AR‐NHL exhibited notable expression of immunomodulatory molecules such as PD‐1 and PD‐L1. Furthermore, patients with dual‐positivity for EBV and PD‐1/PD‐L1 had a poorer clinical outcome. This may be due to several reasons. First, EBV infection has been associated with immunosuppression and chronic antigenic activation, key factors in tumorigenesis. EBV latent infection proteins mechanistically mimic immune‐suppressive cytokines or upregulate PD‐1 in B cells to repress the cytotoxic T‐cell response.^4^ In immunocompetent lymphomas, alterations in PD‐1/PD‐L1 are associated with inferior results, and most EBV‐positive lymphomas show high expression of PD‐1/PD‐L1.^11^, ^18^, ^31^ Second, the notion that HIV lacks oncogenic potential has recently been challenged.^25^, ^32^ PD‐1/PD‐L1 expression in HIV‐specific T cells is associated with T‐cell exhaustion and HIV reservoir formation, which contributes to viral immune evasion and disease progression.^33^

The role of the immune checkpoint signaling axis in virus‐ and immunodeficiency‐associated tumor cell immune evasion is well‐established.^8^ However, knowledge of PD‐1/PD‐L1 expression in NHL is heterogeneous and remains controversial in terms of its prognostic implications.^34^ Our data notably indicated the independent prognostic importance of PD‐1 in patients with AR‐NHL, correlated with strong expression of PD‐L1, EBER^+^, and elevated LDH. When PD‐1, a surface signaling molecule in T cells, binds to the PD‐L1 or PD‐L2 receptors in neoplastic cells and within the tumor microenvironment, it inhibits T‐cell cytotoxicity against malignant cells.^35^ Chronic viral infections accelerate immunosenescence and lead to T‐cell exhaustion, creating an immune‐tolerant environment that can be reversed by PD‐1 blockade.^36^ In fact, PD‐1 and PD‐L1 inhibitors have shown remarkable clinical efficacy in cancer treatment, including hematologic malignancies.^37^ Given the high risk of chemotherapy‐induced comorbidities, treatment with a checkpoint inhibitor could be ideal as an immune‐based approach to improve antitumor immune responses and potentially improve HIV control. In the context of HIV‐related tumors, a recent study using pembrolizumab with or without pomalidomide reported that 6 of 7 patients with EBV^+^ AR‐NHL achieved CR or PR or sustained stabilization of the disease after treatment.^3^ On the other hand, PD‐1 inhibits T‐cell receptor‐induced HIV reactivation in latently infected cells^38^ and may induce prolonged silencing of the HIV promoter via metabolic and epigenetic reprogramming of T cells.^39^ Conversely, PD‐1 blockade triggers transient declines in HIV DNA levels and increases in unspliced HIV RNA, thereby reducing the latent reservoir in vivo.^40^

PD‐L1 expression is a recognized marker of a poor prognosis in aggressive lymphomas, and most EBV^+^ lymphomas are associated with high levels of PD‐L1 expression.^5^, ^9^, ^10^ Our data revealed that 54.2% (28 of 48) of the AR‐NHL tissue specimens evaluated displayed positive expression of PD‐L1. Furthermore, the majority of patients with EBER^+^ AR‐NHL exhibited substantial expression of PD‐L1 (78.6%, Figure 2B). Unfortunately, PD‐L1 did not emerge as an independent prognostic factor in this study, possibly due to the limited sample size. Increasing the sample size or conducting prospective research in the future may help mitigate these limitations and yield more precise conclusions. In HIV‐negative B‐NHL, the main subtype of AR‐NHL, PD‐1/PD‐L1 expression, has been reported to be heterogeneous and has been inconsistently associated with prognosis.^35^ Most patients with classical Hodgkin lymphoma (cHL) have a strong tumor PD‐L1/PD‐L2 expression (70%–87%) because of high frequencies of chromosome 9p24.1 copy number alterations and EBV infection (23%–32%).^34^ However, the expression of PD‐L1 in the context of DLBCL was variable. PD‐L1 was detected in 26%–75% of DLBCL, more frequently in ABC‐DLBCL.^9^, ^11^, ^41^, ^42^ In particular, EBV^+^ DLBCL exhibited higher expression of PD‐L1, although its clinical significance remains unclear. Primary mediastinal B‐cell lymphoma (PMBCL) is characterized by a high rate of PD‐L1/PD‐L2 overexpression mainly associated with the rearrangement of chromosome 9p24.1 (20%).^43^ In chronic lymphocytic leukemia/small lymphocytic lymphoma (CLL/SLL), PD‐L1 expression lacks a significant prognostic value in most studies. In other entities of B‐NHL, the prevalence of PD‐L1 expression in neoplastic B cells is relatively low, and is approximately 5% in follicular lymphoma (FL), 10% in high‐grade marginal zone lymphoma (MZL), and absent in mantle cell lymphoma (MCL).^42^, ^44^, ^45^, ^46^ In addition, more studies are required to accurately assess the importance of the expression of PD‐1/PD‐L1/PD‐L2 in tumor cells and tumor‐infiltrating lymphocytes in the context of AR‐NHL in future research.

Tregs employ various mechanisms, including the PD‐1/PD‐L1 pathway, to maintain self‐tolerance and prevent autoimmunity.^47^ Dysregulation of Tregs in lymphoma can suppress T‐cell and cytokine‐mediated immunity, facilitating tumor dissemination and immune evasion.^48^ However, the expression of PD‐1 on Tregs in AR‐NHL is not clear. Our findings revealed that patients with AR‐NHL with elevated histological expression of PD‐1 also exhibit elevated PD‐1 levels in peripheral blood Tregs, which serves as a supplementary predictor of an inferior prognosis. Additionally, Tregs play a critical role in the balance of immunopathology and antiviral effector responses. Previous studies have indicated that HIV infection leads to an increase proportion of PD‐1^+^ Tregs, correlated with disease progression and HIV‐specific T‐cell exhaustion.^49^ Therefore, further research is necessary to uncover the potential of PD‐1^+^ Tregs as prognostic biomarkers and therapy strategies in AR‐NHL.

This study has several limitations. First, the small sample size limited the statistical power of clinicopathological analyses. Second, the retrospective design led to variations in treatment and follow‐up protocols. Third, the wide spectrum of AR‐NHL subtypes included in our study raises uncertainty regarding the generalizability of the findings across all subtypes. Lastly, the genetic profile of EBV^+^ AR‐NHLs remains unclear. Further research is warranted to elucidate the underlying mechanisms.

In conclusion, our multicenter cohort study revealed a high prevalence of EBV^+^ in patients with AR‐NHL characterized by elevated tissue levels of PD‐1, which is associated with a particularly poor prognosis in the era of chemoimmunotherapy. Notably, PD‐1 was identified as an independent prognostic biomarker in AR‐NHL. These findings underscore the importance of PD‐1‐mediated immune evasion in the complex field of immune–oncology in EBV^+^ AR‐NHLs, pointing to promising avenues for immunotherapy in these patients.

## AUTHOR CONTRIBUTIONS

**Han Zhao:** Conceptualization (equal); data curation (equal); formal analysis (equal); funding acquisition (equal); investigation (equal); methodology (equal); project administration (equal); resources (equal); software (equal); supervision (equal); validation (equal); visualization (equal); writing – original draft (equal); writing – review and editing (equal). **Shaohang Cai:** Investigation (equal); methodology (equal); project administration (equal). **Yanhua Xiao:** Methodology (equal); project administration (equal); resources (equal). **Muye Xia:** Data curation (equal); methodology (equal); project administration (equal). **Hongjie Chen:** Methodology (equal); project administration (equal). **Zhiman Xie:** Data curation (equal). **Xiaoping Tang:** Data curation (equal); methodology (equal). **Haolan He:** Funding acquisition (equal); investigation (equal); methodology (equal); project administration (equal); writing – review and editing (equal). **Jie Peng:** Funding acquisition (equal); investigation (equal); methodology (equal); project administration (equal); writing – review and editing (equal). **Juanjuan Chen:** Conceptualization (equal); formal analysis (equal); funding acquisition (equal); investigation (equal); methodology (equal); project administration (equal); supervision (equal); writing – review and editing (equal).

## FUNDING INFORMATION

This project received funding support from the National Natural Science Foundation of China (No. 81971949), the Clinical Research Program of Nanfang Hospital, Southern Medical University (No. 2018CR026), the GuangDong Basic and Applied Basic Research Foundation (No. 2023A1515030252), and the Municipal and University (Hospital) joint funding project of Guangzhou Municipal Science and Technology Bureau (Nos. 2023A03J0796, 202201020253, and 202201020250).

## CONFLICT OF INTEREST STATEMENT

None of the authors have conflicts of interest to disclose, and none have financial relationships relevant to this article to disclose.

## ETHICS STATEMENT

This research received approval from the Ethics Committees of Nanfang Hospital (NFEC‐2021‐178), Guangzhou Eighth People's Hospital (202210222), and the Fourth People's Hospital of Nanning ([2019]39). All participants provided written informed consent in accordance with the World Medical Association Declaration of Helsinki.

## Supporting information


Data S1.

## Data Availability

The data and material supporting the conclusion are available from the corresponding author upon reasonable request.
