# Supplementary material for: Expression and prognostic significance of the PD‐1/PD‐L1 pathway in AIDS‐related non‐Hodgkin lymphoma
Source: Cancer Med. 2024 Apr 13;13(7):e7195. doi: 10.1002/cam4.7195 (PMC11015146; doi:10.1002/cam4.7195)
Supplement: Supplementary file 1 — Data S1. [file CAM4-13-e7195-s001.docx]

**Expression and prognostic significance of PD-1/PD-L1 pathway in AIDS-related non-Hodgkin lymphoma**

Han Zhao^1, 2†^, Shaohang Cai^1†^, Yanhua Xiao^3†^, Muye Xia^1^, Hongjie Chen^1^, Zhiman Xie^4^, Xiaoping tang^2^, Haolan He^2*^, Jie Peng^1*^, Juanjuan Chen^1*^

^1^Department of Infectious Diseases, Nanfang Hospital, Southern Medical University, Guangzhou, China

^2^Infectious Diseases Center, Guangzhou Eighth People's Hospital, Guangzhou Medical University, Guangzhou, China

^3^Pathology department, Guangzhou Eighth People's Hospital, Guangzhou Medical University, Guangzhou, China

^4^Guangxi AIDS Clinical Treatment Center, the Fourth People's Hospital of Nanning, Nanning, China

*Corresponding author:

Juanjuan Chen

chenjj@smu.edu.cn

Jie Peng

pjie138@163.com

Haolan He

gz8hhhl@126.com

^†^Han Zhao, Shaohang Cai, and Yanhua Xiao shared co-first authorship.**SUPPLEMENTARY FIGURE AND FIGURE LEGENDS**


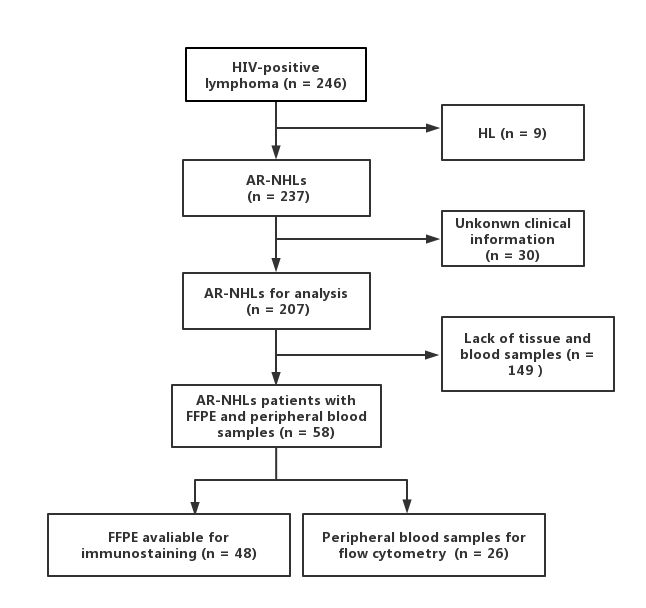


**SUPPLEMENTARY FIGURE 1** Patient distribution flowchart.


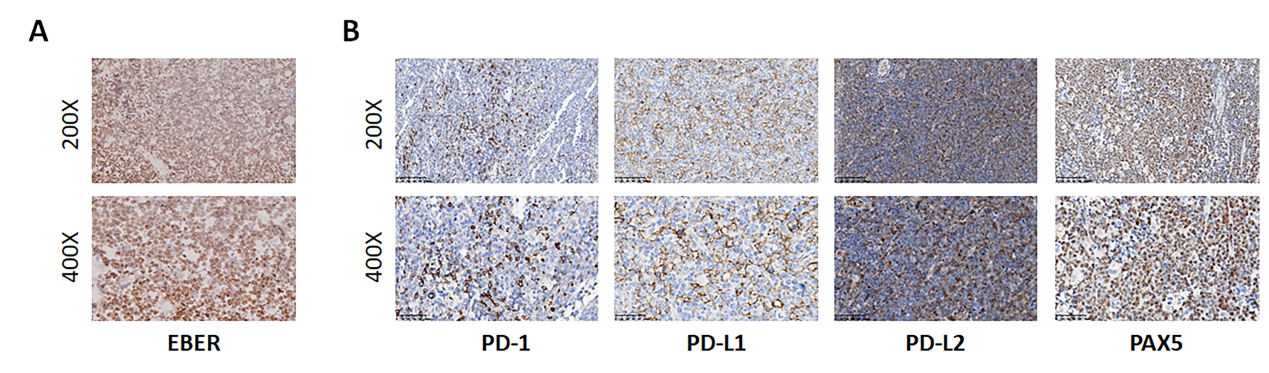


**SUPPLEMENTAL FIGURE 2** Representative morphology of **(A)** EBER in situ hybridization, **(B)** PD-1 and PD-L2 on tumor cells, PD-L1 in tumor cell membranes and/or cytoplasm, and PAX5 in tumor nucleoli.

**SUPPLEMENTARY TABLE 1** The first-line combination anti-retroviral therapy, anti-tumor therapy and response of AIDS-related non-Hodgkin lymphoma patients (*n* = 58).

| **Variable**  (available to evaluate) | **B-cell lymphoma**  (*n* = 55) | **T-cell lymphoma**  (*n* = 3) | **Total**  (*n* = 58) |
| --- | --- | --- | --- |
| **cART** |  |  |  |
| NRTIs + NNRTIs | 26 (47.3) | 2 (66.7) | 28 (41.4) |
| NRTIs + PIs | 2 (3.6) | 1 (33.3) | 3 (5.1) |
| NRTIs + INSTIs | 24 (43.6) | 0 (0) | 24(48.3) |
| Untreated | 3 (5.5) | 0 (0) | 3 (5.2) |
| **Treatment received** |  |  |  |
| Immunochemotherapy ± RT | 35 (63.6) | 0 (0) | 35 (60.3) |
| Chemotherapy ± RT | 9 (16.4) | 1 (33.3) | 10 (17.2) |
| Immunochemotherapy + ASCT | 1 (1.8) | 0 (0) | 1 (1.7) |
| Untreated | 10 (18.3) | 2 (66.7) | 12 (20.8) |
| **therapeutic regimens** |  |  |  |
| CHOP based regimen | 16 (29.0) | 1 (33.3) | 17 (29.3) |
| EPOCH based regimen | 26 (47.3) | 0 (0) | 26 (44.8) |
| Other regimens | 3 (5.4) | 0 (0) | 3 (5.1) |
| Untreated | 10 (18.3) | 2 (66.7) | 12 (20.8) |
| **CNS prophylaxis by intrathecal** |  |  |  |
| Yes | 25 (45.5) | 0 (0) | 25 (43.1) |
| No | 30 (54.5) | 3 (100) | 33 (56.9) |
| **Treatment outcomes** |  |  |  |
| CR | 12 (21.8) | 0 (0) | 12 (20.7) |
| PR | 20 (36.4) | 0 (0) | 20 (34.5) |
| SD | 5 (9.1) | 0 (0) | 5 (8.6) |
| PD | 12 (21.8) | 2 (66.7) | 14 (24.1) |
| Unevaluated | 6 (10.9) | 1 (33.3) | 7 (12.1) |

Abbreviations: cART, combination antiretroviral therapy; NRTIs, nucleoside/nucleotide reverse transcriptase inhibitors; NNRTIs, non-nucleoside reverse transcriptase inhibitors; PIs, protease inhibitors; INSTIs, integrase strand-transfer inhibitors; RT, radiation therapy; ASCT, autologous stem cell transplantation; CHOP, cyclophosphamide, doxorubicin, vincristine, and prednisone; EPOCH, etoposide, vincristine, cyclophosphamide, prednisone, and doxorubicin; CNS, central nervous system; ORR, Overall response rate; CR, complete response; PR, partial response; SD, stable disease; PD, progressive disease.

**SUPPLEMENTARY TABLE 2** International Prognostic Index (IPI) score, c-MYC/BCL2 double-expressor lymphoma (DEL), double-hit lymphoma (DHL), c-MYC/BCL2/BCL6 triple-expressor lymphoma (TEL), and triple-hit lymphoma (THL) of AIDS-related B-cell lymphoma patients (*n* = 55).

| **Variable**  (available to evaluate) | **B-cell lymphoma**  (*n* = 55, %) |
| --- | --- |
| **IPI** |  |
| 0-1 | 24 (43.6) |
| 2 | 4 (7.3) |
| 3 | 10 (18.2) |
| 4-5 | 17 (30.9) |
| **MYC rearrangement** | 5/18 (27.8) |
| **DLBCL** | 2/10 (20.0) |
| **BL** | 3/7 (42.9) |
| **HGBL** | 0/1 (0) |
| **DEL** | 18/36 (50) |
| **DHL** | 3/18 (16.6) |
| **TEL** | 14/36 (38.9) |
| **THL** | 1/18 (5.6) |

Abbreviations: IPI, International Prognostic Index; DEL, double-expressor lymphoma; DHL, double-hit lymphoma; TEL, triple-expressor lymphoma; THL, triple-hit lymphoma.

**Supplementary Table 3** Association of PD-1 expressing T cell subsets in peripheral blood (Multiparameter flow cytometry) and PD-1 expression in tissues (Immunohistochemistry) in AIDS-related non-Hodgkin lymphoma patients (*n* = 16).

| **T cell subsets in PB** | **Immunophenotype** | **PD-1 in tissue** | | |
| --- | --- | --- | --- | --- |
|  |  | **Negative**  **(*n* = 5, %)** | **Positive**  **(*n* = 11, %)** | ***P* value** |
| PD-1^+^CD3^+^ T cells | CD3^+^PD-1^+^ | 5.51 ± 0.94 | 9.94 ± 1.26 | **0.028** |
| PD-1^+^CD3^+^CD4^+^ T cells | CD3^+^CD4^+^PD-1^+^ | 12.84 ± 4.84 | 21.46 ± 3.87 | 0.234 |
| PD-1^+^ inhibited T cells | CD3^+^CD4^+^CD134^-^PD-1^+^ | 7.58 ± 3.05 | 11.79 ± 3.01 | 0.496 |
| PD-1^+^ Tregs | CD3^+^CD4^+^CD25^+^CD127^-^PD-1^+^ | 1.81 ± 0.57 | 5.09 ± 0.93 | **0.041** |
| PD-1^+^ resting Tregs | CD3^+^CD4^+^CD25^+^CD127^-^CD45RA^+^PD-1^+^ | 7.85 ± 4.13 | 4.22 ± 1.30 | 0.421 |
| PD-1^+^ activated Tregs | CD3^+^CD4^+^CD25^+^CD127^-^CD45RA^-^PD-1^+^ | 2.72 ± 1.21 | 4.59 ± 1.05 | 0.396 |
| PD-1^+^CD3^+^CD8^+^ T cells | CD3^+^CD8^+^PD-1^+^ | 14.90 ± 7.55 | 31.18 ± 6.93 | 0.126 |
| PD-1^+^CD3^+^CD8^+^ inhibited T cells | CD3^+^CD8^+^CD134^-^PD-1^+^ | 22.77 ± 8.11 | 39.84 ± 6.94 | 0.193 |

Abbreviations: PB, peripheral blood.

**SUPPLEMENTARY TABLE 4** CD4^+^ T cell levels during and after first-line chemotherapy in AIDS-related non-Hodgkin lymphoma individuals (*n* = 58).

| **Variable**  (available to evaluate) | **CD4 count (cells/μL)**  (median, IQR) |
| --- | --- |
| At initial lymphoma diagnosis | 182 (92–304) |
| 2 chemotherapy cycles | 196 (81–354) |
| 4 chemotherapy cycles | 168 (48–297) |
| 6 chemotherapy cycles | 142 (81–245) |
| 12 weeks after the end of chemotherapy | 215 (103–327) |
| 24 weeks after the end of chemotherapy | 340 (185–432) |
| 36 weeks after the end of chemotherapy | 329 (164–406) |
| 48 weeks after the end of chemotherapy | 341 (189–475) |
| 72 weeks after the end of chemotherapy | 331 (172–453) |
| 96 weeks after the end of chemotherapy | 374 (172–479) |

Abbreviations: IQR, interquartile range.

**SUPPLEMENTARY METHOD 1**

Tissue samples from different patients or experimental conditions are carefully selected to ensure representation. Thin sections of representative tissues are taken from the donor blocks using a microtome and mounted onto a recipient block, made of paraffin embedding medium. Small cylindrical tissue cores, typically 0.6-2 mm in diameter, are obtained from the donor blocks and precisely arrayed onto the recipient block. The recipient block containing multiple tissue cores is heated to allow fusion and adhesion of the cores to the block, resulting in a compact TMA block.

In brief, TMA were sectioned continuously at a thickness of 4 μm and heated for 1 h at 60℃，the sections were then deparaffinized using xylene at 37℃ for 20 min and rehydrated with a series of graded alcohol and distilled water. The tissue slides were then treated with 3% hydrogen peroxide in methanol for 20 min at 37℃ to block endogenous peroxidase activity. Then processed for antigen retrieval by high pressure cooking in EDTA antigen retrieval solution (pH = 9) for about 2.5 minutes and allowed to cool to room temperature. Followed by incubation with rabbit monoclonal anti-PAX5 (Abcam, #ab109443, dilution 1:500), rabbit monoclonal anti-PD-1 (Abcam, #ab214421, dilution 1:100) and rabbit monoclonal anti-PD-L2 (Abcam, #ab288298, dilution 1:1000), in a humidified container overnight at 4℃. The tissue slides were washed three times with PBS, incubated with the corresponding secondary anti-bodies anti-rabbit/mouse (PV-6000, Zhongshan Goldenbridge Biotechnology Co., China), at 37℃ for 30 minutes then thoroughly washed three times with PBS. The sections were developed with diaminobenzidine tetrahydrochloride (DAB) and counterstained with hematoxylin. PD-L1 IHC slides were performed on the Dako Omnis according to the manufacturer’s instrument specific labeling protocol with the PD-L1 22C3 antibody. Human tonsil served as positive controls for the staining of PAX5, PD1, PD-L1, and PD-L2.

For PD-L1, our study employed the combined positive score (CPS) to provide a comprehensive assessment of membrane staining positivity on tumor cells, directly correlated with the presence of lymphocytes and macrophages within the tumor microenvironment. CPS = PD-L1 membrane staining positive tumor cells + PD-L1 membrane staining positive tumor related immune cells (lymphocytes and macrophages)/total tumor cell count × 100.^1^  PD-L2 evaluation was identical with PD-L1.^2^

In NHL, TILs expressed PD-1, contrasting with PD-L1 expression on TCs and TILs. Thus, the numbers of PD-1 cells were assessed semiquantitatively or percentage based on the previous study.^3, 4^ In our study, a positive score was assigned to PD-1 staining in over 5% of TIL cells.^3^

**SUPPLEMENTARY METHOD 2**

Briefly, PBMCs were thawed, washed and directly stained ex vivo using a Live/Dead Zombie Fixable Viability Kit (BioLegend, San Diego, CA) for 10 min at room temperature to exclude nonviable cells. They were then washed and stained with anti-human CD45RA-BV785, CD3-APC/Cy7, CD4-FITC, CD8-PE/Cy7, CD25-PE, CD127-BV421, CD134-APC, and CD279 (PD-1)-BV650 (all BioLegend, San Diego, CA), for 30 min in the dark at 4℃, followed by washing twice. The supernatant was discarded, and re‐suspended in 0.2 mL PBS.

**References:**

1.Shah MA, Kennedy EB, Alarcon-Rozas AE, et al. Immunotherapy and targeted therapy for advanced gastroesophageal cancer: ASCO guideline. *J Clin Oncol* (2023) 41(7): 1470–1491.

2.Gu Q, Li J, Chen Z, et al. Expression and prognostic significance of PD-L2 in diffuse large B-Cell lymphoma. *Front Oncol* (2021) 11(664032.

3.Yang K, Xu J, Liu Q, Li J, Xi Y. Expression and significance of CD47, PD1 and PDL1 in T-cell acute lymphoblastic lymphoma/leukemia. *Pathol Res Pract* (2019) 215(2): 265–271.

4.Kwon D, Kim S, Kim PJ, et al. Clinicopathological analysis of programmed cell death 1 and programmed cell death ligand 1 expression in the tumour microenvironments of diffuse large B cell lymphomas. *Histopathology* (2016) 68(7): 1079–1089.
